# Chess Not Checkers: Complexities Within the Myeloid Response to the Acute Kidney Injury Syndrome

**DOI:** 10.3389/fmed.2021.676688

**Published:** 2021-05-26

**Authors:** William T. Nash, Mark D. Okusa

**Affiliations:** Division of Nephrology, Department of Medicine, Center for Immunity, Inflammation, and Regenerative Medicine, University of Virginia, Charlottesville, VA, United States

**Keywords:** macrophages, monocytes, F4/80, neutrophils, acute kidney injury, cell death, inflammation

## Abstract

Immune dysregulation in acute kidney injury (AKI) is an area of intense interest which promises to enhance our understanding of the disease and how to manage it. Macrophages are a heterogeneous and dynamic population of immune cells that carry out multiple functions in tissue, ranging from maintenance to inflammation. As key sentinels of their environment and the major immune population in the uninjured kidney, macrophages are poised to play an important role in the establishment and pathogenesis of AKI. These cells have a profound capacity to orchestrate downstream immune responses and likely participate in skewing the kidney environment toward either pathogenic inflammation or injury resolution. A clear understanding of macrophage and myeloid cell dynamics in the development of AKI will provide valuable insight into disease pathogenesis and options for intervention. This review considers evidence in the literature that speaks to the role and regulation of macrophages and myeloid cells in AKI. We also highlight barriers or knowledge gaps that need to be addressed as the field advances.

## Introduction

Acute kidney injury (AKI) is exemplified by a disruption in renal homeostasis that leads to a rapid decrease in kidney function. This is a devastating condition that continues to lack effective therapies. The combination of severe health impairment and high prevalence creates a significant burden for both patients and health systems. Patients with AKI have higher hospitalization costs, longer hospital stays, decreased quality of life, and an increased risk of death compared to non-AKI patients (1–4). A 2013 meta-analysis concluded that 1 in 5 adults and 1 in 3 children develop AKI during a hospital stay (5); currently, it is estimated that ~500,000 hospitalized individuals are affected by AKI in the U.S. alone (1). This is clearly a widespread and pervasive threat to human health that must be addressed.

Cell death following injury is a key event in AKI initiation (2, 6, 7). Tissue damage and cellular stress leads to the release of molecules and byproducts with a wide range of potential effects on surrounding cells. It is therefore important to understand how specific, well-defined cell populations respond to cues within the microenvironment and participate in AKI pathogenesis, since such knowledge can inform and improve intervention strategies.

Cellular death can occur via multiple pathways, including regulated mechanisms (e.g. apoptosis, necroptosis, pyroptosis, ferroptosis) or unregulated, accidental necrosis (7–11). The extent and type of cell death within injured tissue has important consequences for downstream responses, the content of the microenvironment, and ultimate outcome (12). Generally, apoptosis is considered a well-contained event that minimally affects surrounding cells and promotes homeostasis. Other forms of cell death, however, can trigger immune cell activation and inflammatory pathways, leading to extension of the original injury (7–14).

Inflammatory events downstream of cellular death in AKI is an area of ongoing investigation, but fully understanding these cascades will be instrumental for improving therapy (6). While the decline in kidney function during AKI stems from damage to epithelial cells and loss of tubule function, immune cells play important roles in early and late phases of AKI (2, 6, 15–21). The increased presence of myeloid cells has been widely reported and is considered a key event in the pathogenesis of AKI (2, 6, 15–21). Myeloid cells (e.g., macrophages, monocytes, neutrophils) can disrupt the structural integrity of tissue and produce molecules that are toxic to surrounding cells, such as cytokines, reactive oxygen or nitrogen species, and purines. These activities can extend the initial injury and potentially exacerbate the severity of AKI. Later in the course of AKI, myeloid cells are also capable of delaying resolution and recovery by sustaining inflammation and vascular impairment (10, 16, 22).

Macrophages, in particular, are equipped with an extensive array of danger and cytokine sensing receptors for surveying their surroundings (23, 24). They are exquisitely sensitive to changes in the microenvironment and can adopt a spectrum of activation states in response to environmental cues (25–27). Macrophages are the major immune population present in healthy kidneys and have the capacity to participate in many aspects of AKI pathogenesis (2, 6, 15, 16, 20, 21, 28). They have diverse functions in tissue which include engulfment of debris and damaged cells, detection of danger and damage via pattern recognition and cytokine receptors, production of cytokines and oxygen/nitrogen species, destruction and deposition of tissue matrix, and recruitment of additional immune cells (29). Important concerns regarding the general complexity of macrophage differentiation and activation states are beyond the scope of this discussion, but have been nicely addressed elsewhere (25, 27, 30, 31). Due to the controversy surrounding this topic and M1/M2 nomenclature, we will focus on the regulation of macrophages and myeloid cell populations during AKI rather than classification of activation states.

The role of macrophages as sentinels of the tissue environment and the fact that they are the pre dominant immune population pre-injury makes them prime candidates for further study in the context of AKI pathogenesis. A clearer understanding of the relationships between tissue damage, macrophage activation, inflammation, myeloid accumulation, and injury progression holds promise for the development of novel therapies for AKI. Here we will discuss the potential involvement of macrophages and key myeloid populations in AKI as well as some barriers that have generated confusion in the context of the kidney.

## Cell Death and Macrophages

Cell death is a key element in the initiation of injury and reduced renal function in AKI (2, 6, 7). As stated above, cell death can manifest via multiple mechanisms including apoptosis, necrosis, necroptosis, pyroptosis, and ferroptosis (8, 9). Broadly, apoptosis is considered an immunologically silent process that does not result in inflammation, although this may not always be the case (8). Currently, there is some controversy surrounding the extent of the role of apoptosis in AKI (7). While heightened apoptotic death of tubule cells can be an important element of initial injury and loss of renal function, current knowledge suggests it is unlikely that this death mechanism contributes extensively to downstream inflammatory responses in AKI. On the other hand, cell death stemming from necrosis, necroptosis, pyroptosis, or ferroptosis can function as an important initiator of macrophage and myeloid cell activation, representing a potential bridge from initial injury to pathogenic inflammation (9, 10, 13, 32).

### Cell Death Mechanisms and Inflammation

Apoptosis is a programmed form of cell death that occurs during homeostatic turnover, accumulation of cellular stress and damage, or during immune recognition of infected or improperly functioning cells. This process hinges on the ultimate activation of executioner caspases 3 and 7 and multiple intrinsic and extrinsic pathways can trigger this outcome (8). Generally, apoptosis proceeds after controlled permeabilization of the outer membrane occurs without fully disrupting its integrity. Following initiation of apoptosis, the cell undergoes controlled dismantling and fragments into membrane-encapsulated apoptotic bodies. This prevents mass-escape of cellular contents into the surrounding environment and allows the apoptotic material to be removed without inducing excessive local inflammation. The clearance of apoptotic cells and the apoptotic bodies they produce is referred to as efferocytosis.

Inflammatory forms of cell death lead to the release of intracellular components and activation of extracellular molecules that can be interpreted as danger signals and potentially shift macrophages from a maintenance phenotype to an inflammatory phenotype (9, 24, 26, 33). Un-programmed, accidental necrosis can occur when cells are suddenly and irreparably damaged and can no longer maintain their structural integrity. Induction of necrosis can result from direct tissue trauma or drastic changes in the environment, such as severe hypoxia, removal of growth factors, or depletion of cellular ATP (8, 12). Necrosis can also occur downstream of apoptosis. If apoptotic cells cannot be cleared and processed and are unable to return to a homeostatic state, they will progress to secondary necrosis (12). This mechanism could be at play in injured kidneys if the degree of apoptosis surpasses the efferocytic capacity of the tissue. Necrotic cell death is characterized by a loss of cell membrane integrity leading to swelling and eventual rupture and the unregulated release of cellular contents.

While necrosis is the only described form of unregulated cell death, the necrotic pattern of cell death (i.e., the release of cellular components) can also manifest as the result of programmed cellular execution mechanisms. Each of these mechanisms culminates in destabilization of the cell membrane and the release of toxic or inflammatory cellular contents. During necroptosis, association of RIPK1 and RIPK3 leads to activation of the executioner protein mixed lineage kinase domain-like (MLKL) (8, 9, 12, 32, 34). MLKL goes on to form pores in the membrane and precipitate necrotic death. In pyroptosis, the executioner gasdermin proteins (gasdermin-D is the most commonly studied to date) are cleaved into their active form by caspases. The active fragment of gasdermins, similar to the role of MLKL, oligomerizes and forms pores in the membrane (8, 12, 32). Ferroptosis is a distinct form of cell death that is dependent on iron availability and occurs as the result of increased lipid oxidation (8, 9, 12, 32, 35). Iron promotes lipid oxidation either directly via Fenton reactions or indirectly as a component of enzymes, such as lipoxygenases (LOX) (8, 36, 37). Since lipids are a major component of cell membranes, unrestricted lipid oxidation within cells is extremely damaging to their structural integrity and leads to rapid necrotic death (8, 32, 35, 36, 38). All of these mechanisms are capable of creating an environment that favors inflammation over resolution of injury.

### Cell Death as the Bridge Between Injury and Inflammation

**Figure 1:**
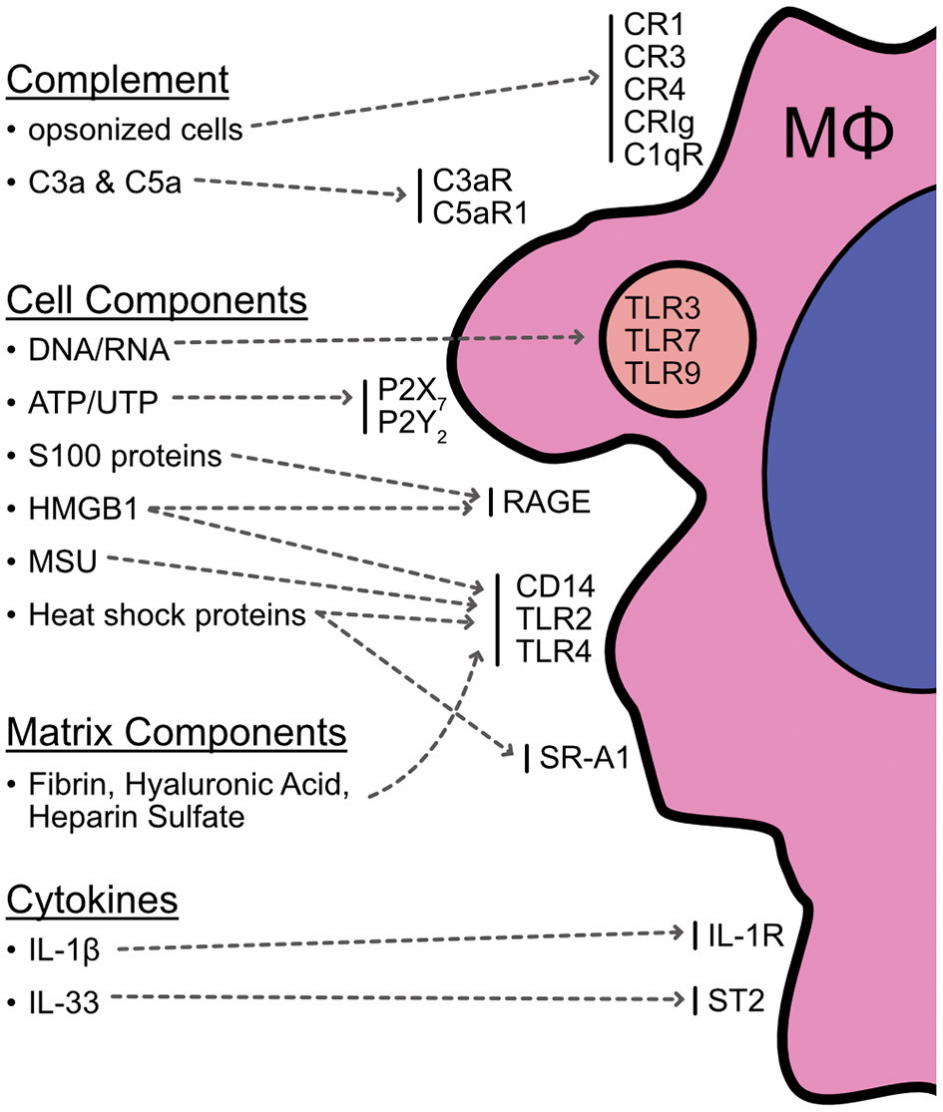
Summary of potential interactions between macrophages and cell death. Macrophages express a variety of machinery for detecting cell death and products released from cells or the surrounding matrix as a result of necrotic death. These include receptors for complement opsonized cells (CR1, CR3, CR4, CRIg, C1qR); complement cascade components (C3aR, C5aR); DNA/RNA (endosomal TLRs 3, 7, & 9); purines (P2X_7_, P2Y_2_); monosodium urate (CD14/TLRs); S100 proteins (RAGE); HMGB1 (RAGE, TLRs); heat shock proteins (TLRs, SR-A1); the matrix components fibrin, hyaluronic acid, and heparin sulfate (TLRs); and cytokine alarmins such as IL-1β and IL-33 (IL-1R, ST2). This list is not exhaustive and additional interactions are continuously being described and investigated. It is still unclear to what extent these interactions are involved in AKI and if macrophage stimulation is a critical element for bridging injury and pathogenic inflammation, but it is clear there are multiple avenues for macrophages to engage with cell death events in a tissue setting.

While the identity and activity of pro-inflammatory mediators produced during cell death are still under investigation, the list of culprits includes DNA and DNA-chromatin complexes, heat shock proteins (HSP), high-mobility group box 1 (HMGB1), uric acid, galectins, purines (e.g., ATP), extracellular matrix components, and complement system activation (13, 39). In addition, cytokines in the IL-1 family can act as inflammatory danger signals during cell death, including IL-1β and IL-33 (40). The bioactivity of these cytokines is generally proteolytically regulated. Caspase activity can variably increase or dampen the ability of these cytokines to stimulate inflammation, so the array of cytokine activity will depend on the mechanism of cell death. For example, full-length IL-1β does not bind the IL-1 receptor and requires cleavage by caspase-1 (or potentially other enzymes) to exert its activity. Conversely, active IL-33 is sequestered within cells and is inactivated by caspases 3 and 7 (40). Caspase-independent cell death can therefore bypass this inactivation and lead to the release of active, pro-inflammatory IL-33 that acts as an alarmin and induces inflammation (40). Macrophages express receptors that can detect a majority of these damage-associated molecules and are prime candidates for shaping the immediate response following initial injury (Figure 1). Macrophage receptor machinery and responses to cell death have been nicely reviewed elsewhere (9, 13, 23, 24, 41), but these interactions will be briefly explored here.

In addition to the above-mentioned forms of cell death, NETosis can also play a role in AKI. This is a form of death specific to neutrophils upon the release of neutrophil extracellular traps (NETs) (32, 42, 43). NETosis occurs when neutrophils are triggered to release mesh-like structures of their intracellular components comprised of DNA, histones, and granule proteins (32, 42, 43). While this process is specific to neutrophils and NETosis is not a form of death that occurs in tissue cells, the release of these intracellular components forms a milieu reminiscent of other forms of necrotic cell death (32, 42, 43). Thus, NETosis can also participate in cell death-induced inflammation and thrombosis during AKI.

Complement can be deposited on the surface of dead/dying cells and apoptotic bodies to facilitate recognition and uptake by macrophages via complement receptors (CR). These include CR1 (CD35), CR3 (CD11b/CD18), CR4 (CD11c/CD18), CRIg, and C1qR (CD93) (24, 44). Clearance of dead/dying material is generally beneficial during injury and appears more suppressive than stimulatory for macrophages (13, 24, 41). Thus, this may be a means of attempting to limit inflammation in the face of cell death. However, in addition to this interaction with dead/dying cells, macrophages can also detect the C3a and C5a products of the complement cascade via C3aR and C5aR1 (CD88) (44). While detection of these components activates macrophages and can stimulate the production of proinflammatory cytokines (45), there is also evidence that they can suppress macrophage functions and promote tumor growth or metastasis (46–50). Given this, the impact of complement on macrophages is likely context dependent and dictated by the extent and type of cell death as well as additional stimuli experienced by macrophages. Interestingly, a study by Peng et al. has shown that C3aR and C5aR deficiency can protect mice from renal ischemia reperfusion injury (IRI) (51), which shows these receptors play a role in pathogenesis. However, since the genetic deficiency was not restricted to macrophages, it is still unclear if the impact of complement signaling was due specifically to macrophages sensing these molecules. The relationship between macrophages and complement during tissue injury is clearly complex and is further obfuscated by the fact that a wide variety of cells respond to complement components. It will require careful study to dissect the importance of complement for macrophage function relative to other cells during AKI pathogenesis.

Once necrotic death has occurred and cell contents have been released into the environment, macrophages sense these components through a variety of receptor machinery. The toll-like receptor (TLR) family of receptors is expressed extensively by macrophages and may play an important role in the macrophage response to cell death, similar to its role in pathogen detection (13, 24, 52, 53). Members of the TLR family (TLR2 and TLR4) can bind HSP, HMGB1, fibrin, hyaluronic acid, and heparan sulfate (13, 24). These molecules are released from the dead cells themselves (HSP, HMGB1) or generated by the breakdown of extracellular components (fibrin, hyaluronic acid, heparin sulfate) by enzymes released from dead/dying cells (13). TLR can also recognize DNA and RNA released from dead cells (TLR3, TLR7, TLR9) (13, 24). There is also evidence for the stimulation of TLR2/TLR4 by uric acid (also referred to as monosodium urate; MSU), the final cellular product from the breakdown of purines (13, 54). CD14 is another pattern recognition receptor that associates with TLR2 and TLR4 and has been experimentally shown to directly bind MSU, indicating a potential means of MSU recognition, internalization, and cellular activation via CD14/TLR cooperation (13, 55). However, there is still controversy surrounding the stimulatory mechanisms of MSU and its crystal form since others have observed a response to MSU independent of TLR (13, 56).

In addition to TLR and TLR-associated proteins, other macrophage receptors are capable of engaging with products of cell death. The scavenger receptors RAGE and SR-A1 detect HMGB1, S100 proteins, and HSP (13, 24). P2X_7_ and P2Y_2_ receptors bind extracellular ATP and UTP and can trigger macrophage activation (13, 24, 57, 58). Macrophages also express the IL-1 receptor and ST2 and can be stimulated by IL-1β and IL-33 (respectively) (24, 59, 60). This is not a comprehensive list of the interactions that can occur between macrophages and the products of cell death and there are likely additional mechanisms yet to be described, but it is clear there is ample opportunity for engagement and stimulation of macrophages in an environment of widespread tissue damage and cell death.

## The Ambiguity of F4/80

**Figure 2:**
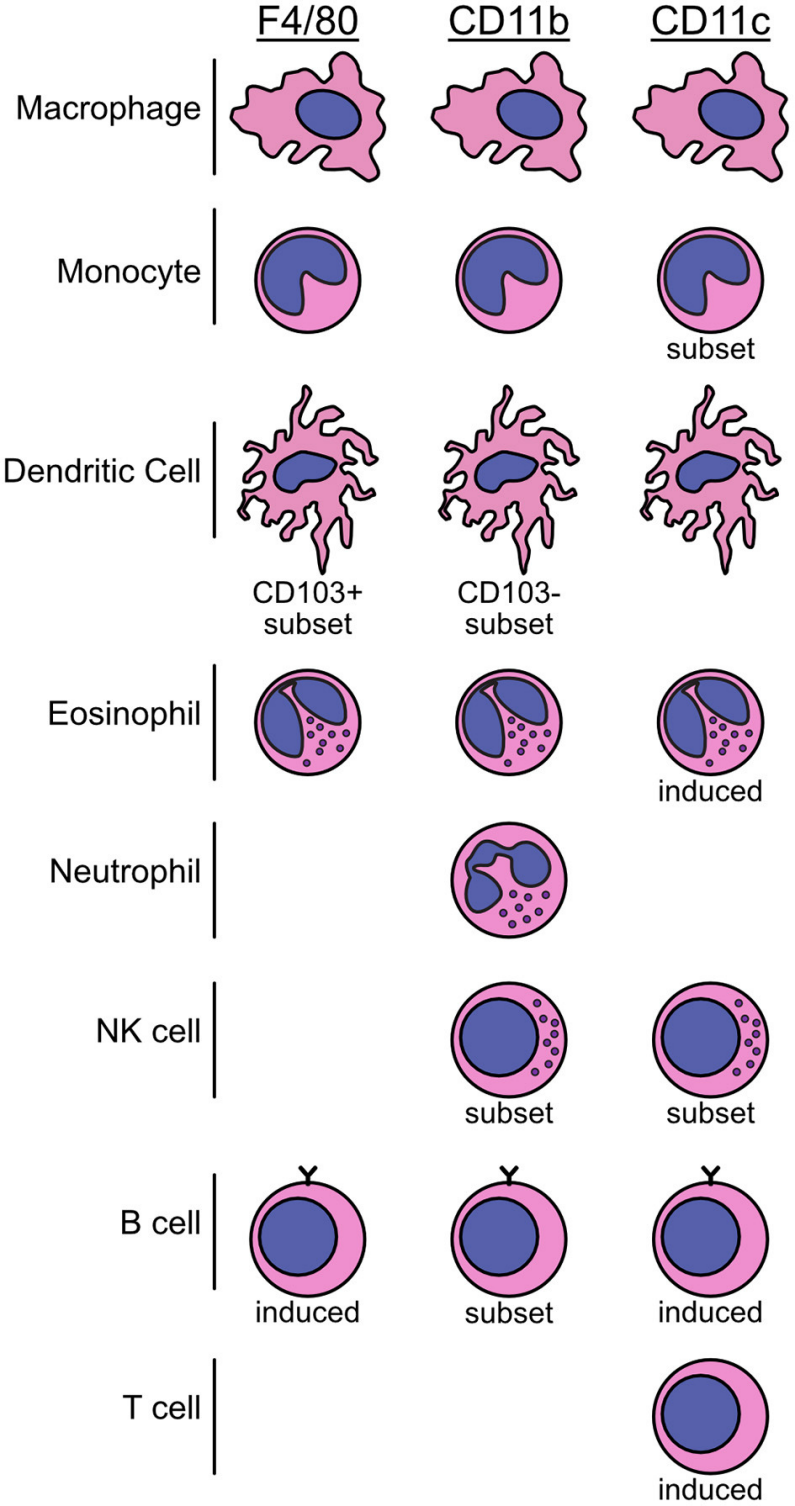
Summary schematic of immune cell populations in mice that express the F4/80, CD11b, and CD11c markers. The use of F4/80, CD11b, and CD11c markers to specifically detect or deplete macrophages has led to a lack of clarity in the AKI field. These molecules are not restricted to macrophages, monocytes, or DC and can be expressed by a variety of immune cell populations and subsets. Further, they can be expressed basally or be induced on specific cell types. Over-reliance on these three molecules without additional characterization can lead to uncertainty about which cells are responsible for an experimental outcome, hindering the ability to draw precise conclusions. The overlapping, diverse expression patterns of these markers is illustrated schematically here.

While F4/80 has traditionally been used as a lineage marker for macrophages, expression of this molecule is far from definitive or exclusive. In reality, many cells express the F4/80 antigen, either basally or as an induced marker. This list includes macrophages, monocytes, DC, eosinophils, and potentially some B cell populations (Figure 2) (61–68). Such non-selectivity can be problematic when attempting to make conclusions about renal macrophages based solely on F4/80 staining in tissue sections, a common occurrence in older literature.

Recent work has shed light on more refined strategies for separating mononuclear phagocyte cell (MPC) populations within the kidney and shows promise for enhancing our sophistication and accuracy when investigating these cells in the context of AKI. In addition to F4/80, flow cytometry studies have shown that CD11b, CD11c, CX3CR1, Ly6C, CD64, CD14, CD16, MHC II, and CD103 are also useful markers for delineating myeloid populations within the mouse kidney (Table 1) (21, 69–80). A study from Lee et al. used several of these markers to identify a novel MPC population in the kidney that is also CD45-intermediate (CD45^int^ CD11b^int^ F4/80^+^ MHCII^+^ CX3CR1^+^ Ly6C^−^). This population is more sensitive to depletion with clodronate than other kidney MPC, but their precise role in kidney injury is still unclear (81). While progress has been made in defining myeloid subsets, the populations identified using these markers still represent macrophages, monocytes, and dendritic cells to varying degrees and each of these cell types contain additional subpopulations. Care must be taken to properly classify these subsets and their functions as the field progresses. Investigators should always be deliberate when describing the populations they are studying and explicit when conveying conclusions that can or cannot be drawn from their work.

**Table 1:** Potential surface markers for classifying renal MPC populations in mice.

	**Macrophages**	**Monocytes**	**Dendritic cells**
CD11*^a^, ^b^*	**++/+++**	**+++**	+/+++^(**)^
CD11c	**+/++**	**++** **(subset)**	**+++**
CD14	**+++**	**++**	**++**
CD16	**++**	**–/+**	**++/+++**
CD64	**++**	**–/+**	**–**
CD68	**+++**	**++**	**–**
CD103	**–**	**–**	**+++** **(subset)**
CD115 ^(*)^	**–**	**–/++**	**–**
MHC II	**+/++**	**++**	**+++**
F4/80	**+++**	**++**	**–/+** **(subset)**
CX3CR1	**++**	**++**	**++**
Ly6C	**–**	**+++** **(subset)**	**–**
Ly6G	**–**	**–**	**–**

a *Marker expression compiled from sources cited in the text and personal experience*.

b *-, not detectable; +, low expression; ++, intermediate expression; +++, high expression; –/+, range of expression from negative to low; +/++, range from low to intermediate; ++/+++, range from intermediate to high*.

(*) *CD115 expression may be variable between blood and tissues and is susceptible to loss in some conditions, so may not be consistently reliable*.

(**) *The two identified DC subsets in kidney have differential CD11b expression. CD103+ DC have low expression while the other subset exhibits high expression*.

*Subset means the expression is found on a defined sub-population of the cell type and not on the cell population as a whole*.

## Collateral Damage in Depletion Strategies

Depletion studies are a powerful tool for attributing phenotypes to specific cell populations. For investigating myeloid cells in AKI, the most common depletion models encountered in the literature are injection of liposome encapsulated clodronate to deplete phagocytic cells or injection of diphtheria toxin (DT) to deplete cells that specifically express the diphtheria toxin receptor (DTR) under control of CD11b or CD11c regulatory elements. However, these models often contain pitfalls that may not be fully accounted for in all studies. A particular concern when investigating myeloid populations stems from the large degree of marker and functional overlap within the myeloid compartment (82–84). Thus, results from depletion studies can compound confusion generated by imprecise cell type classifications. In addition, different depletion models or the same depletion strategy used in different disease models may produce conflicting results. This is readily evident in AKI investigations and has hindered the ability to draw clear conclusions in some cases.

Clodronate is a bisphosphonate compound that is converted into a toxic ATP analog within cells. The toxic product of clodronate metabolism interferes with processes critical to cell survival and triggers subsequent apoptosis (85). Encapsulation of clodronate in liposomes targets its uptake to phagocytic cells. While this is touted as an efficient means of depleting macrophages, macrophages are not the only phagocytic cells. Despite its ability to remove macrophages, clodronate also efficiently depletes DC and monocytes (86–88). Whenever a depletion strategy is used, best practice should be to show the impact of the treatment on additional cell populations, not just the cells of interest. Generally, clodronate experiments should be supported by additional evidence and/or additional depletion models to specifically implicate macrophages, DC, or monocytes.

The DTR has been identified as the heparin-binding EGF-like growth factor (HB-EGF) precursor (89). Although mice express this molecule, key sequence differences greatly impair or prevent the binding of DT and render normal murine cells insensitive to DT-induced death and depletion. This dichotomy allows the engineering of mice that transgenically express the human or simian form of the DTR in specific cell populations (90, 91). This can be done with a DTR transgene under direct transcriptional control of a specific promoter or via a Cre-lox system. In the Cre-lox version, expression of the Cre recombinase enzyme is under control of a specific gene promoter (e.g., CD11b or CD11c promoters) that restricts its activity to a specific cell population. Cre activity in these cells excises a lox-p-flanked STOP codon preceding the DTR gene, allowing transcription, and expression of the DTR to proceed (92).

Similar to the clodronate depletion model, CD11b- or CD11c-dependent DTR expression is not sufficient to attribute outcomes specifically to macrophages or DC (93). CD11b is widely expressed within the myeloid compartment and is variably expressed by tissue macrophage populations. Monocytes, neutrophils, and eosinophils all express high levels of CD11b and some natural killer (NK) cell and DC subsets express this marker as well (Figure 2) (21, 94–97). Lung macrophages express low levels of CD11b while splenic and renal macrophages express low-mid levels and peritoneal macrophages express high levels of the marker (98, 99). CD11c is a ubiquitous DC marker, but it is also expressed by macrophage populations, monocyte subsets, some NK cells, and can be induced in many others (Figure 2) (96, 97, 100–105). The non-specificity of CD11b and CD11c models should lead to a reduction in their use in favor of more specific depletion models in the future.

The complexity of these depletion strategies is readily evident in the AKI literature. Multiple studies have shown that pretreatment with liposome encapsulated clodronate can lessen initial injury in ischemic AKI models (106–109). However, this protection is not observed when depletion is performed with CD11b-DTR or CD11c-DTR models (107, 108). This was nicely shown by Ferenbach et al. (107) and Lu et al. (108) in studies where they compared the effects of clodronate and CD11b-DTR depletion. These investigations noted multiple interesting differences between the models. Clodronate treatment resulted in substantial depletion of kidney F4/80+ cells and blood monocytes, but DT treatment resulted in more complete depletion of both (107). Clodronate also depleted F4/80+ cells in the spleen and liver while DT treatment left these populations essentially intact (107). Pretreatment with clodronate lessened the increase in serum creatinine and tubule injury after AKI, but DT pretreatment did not exhibit the same benefit (107, 108). Further, when clodronate and DT were given in combination, the protection seen with clodronate alone was negated and injury restored to the level seen in control mice (107). The implication here is that the myeloid compartment contains both beneficial and detrimental populations that are active during AKI. While clodronate appears to remove an injury-promoting population, the more extensive depletion from CD11b-DTR also removes cells that are involved in ameliorating injury. This is promising data that requires additional investigation to determine the relevant cells and their functional responses. The impacts of CD11c-DTR depletion, however, are somewhat unclear as conflicting results have been observed showing either minimal impact or some degree of protection in the IRI setting (108, 110).

As stated above, the CD45^int^ MPC population identified by Lee et al. was more sensitive to clodronate depletion than other kidney MPC populations, making these cells an interesting candidate for a pathogenic population (81). At steady state, they were more phagocytic than their CD45^hi^ counterparts but this profile flipped during IRI with CD45^hi^ MPC exhibiting stronger phagocytic activity (81). The CD45^int^ MPC were not significant producers of cytokines during IRI, but this may require additional studies to confirm (81). The contributions of this population at steady state and during injury are still enigmatic and warrant additional study, especially given that they were identified in human samples as well as mice.

While clodronate treatment prior to injury appears beneficial in ischemic AKI, this treatment offers minimal protection in other AKI models such as cisplatin or DT-induced death of DTR-expressing tubule cells (111–114). In fact, macrophage depletion in these models is sometimes associated with worse or prolonged disease. CD11c-DTR depletion in particular exacerbates disease during cisplatin-induced injury (114). It is unknown what causes the divergent effects of depletions in different AKI models, but differences in the mechanism of injury initiation could play a role (inflammation-mediated cell death vs. direct nephrotoxicity). The environment generated by complete ischemia is very different from the environment during targeted nephrotoxicity, and thus the involvement of myeloid cells may vary between models and stages of pathogenesis.

## The Pathogenic Importance of Myeloid Infiltration of Injured Kidneys

The ability of macrophages to recruit additional immune cells is a key area of interest in AKI. The increased representation of neutrophils and F4/80+ cells in the kidney is frequently associated with disease progression and pathogenic inflammation (16, 28). However, the exact contribution of these infiltrating cells to disease is an unsettled issue. Studies that investigate these phenomena often report conflicting results and this has hindered the ability to draw clear conclusions and develop therapeutics.

### Neutrophils

Neutrophils have been investigated in multiple AKI models with varying results, but IRI studies are most prevalent in the literature. Early studies with rats and rabbits indicated that neutrophil depletion strategies produced no significant relief from kidney IRI, aside from reducing the degree of leakage observed in renal tubules (115–117). Another study in rats, however, observed that inducing neutropenia did indeed result in lower creatinine levels and injury scores post-IRI (118). Despite these incongruous results, the fact remains that neutrophils are recruited early and in large numbers during kidney injury and interest in their involvement has persisted.

A variety of mouse studies have now been performed that also speak to the role of neutrophils in AKI. A 2002 study reported that, while pan-caspase inhibition was capable of preventing ischemic injury by reducing cell death, neutrophil depletion produced only a mild benefit to serum creatinine levels and no improvement in acute tubule necrosis (ATN) scores (119). However, several others have shown that interfering with molecules that regulate trafficking and tissue infiltration (ICAM-1, P-selectin, E-selectin, Slp76, ADAP) can limit neutrophil recruitment to the kidney and lessen injury severity (120–122). Interrupting the inflammatory response during injury via adenosine receptor agonism, preventing NKT cell activation, or disrupting an IL-17/INFγ signaling axis can also restrict the influx of neutrophils during IRI and limit injury (18, 19, 123). The biggest issue with these observations is that these types of interventions affect immune cell function in a relatively broad manner and do not specifically target neutrophils. However, several of these studies bolstered their results by including neutrophil depletions that also exhibited reduced injury (120, 121, 123). It remains to be seen if differences between previous work and more recent mouse studies are due to species or procedural differences, but the preponderance of evidence indicates a role for neutrophil involvement in ischemic AKI.

Other AKI models have also reported a benefit to neutrophil depletion. In endotoxemia/sepsis-induced AKI, several studies have reported that neutrophil depletion can limit creatinine increases and kidney injury markers (124–126). Neutrophil depletion in the context of mercuric chloride-induced kidney injury also prevented increases in BUN to a large degree (127). When cholesterol crystals were used to induce renal infarcts and kidney injury, neutrophil depletion improved all injury measurements except glomerular filtration rate (GFR) (128). This is an interesting outcome since GFR is the most relevant measurement for kidney function; however, the reduced GFR was due to obstruction caused by the cholesterol crystal clots, an aspect in which neutrophils may have a minimal or redundant role. Thus, this could be interpreted as neutrophil depletion preventing additional inflammation and injury that is secondary to the initial injury caused by the obstructions.

Cisplatin-induced AKI, however, stands apart from other injury models. Neutrophil depletion in this context has repeatedly failed to produce a benefit. One study noted an association of increased IL-1β, IL-18, IL-6, and neutrophil infiltration with cisplatin-induced injury. However, when the activity of the cytokines was inhibited or removed or neutrophils were depleted, there was no reduction in kidney injury (129). Another study also observed that neutrophil depletion failed to prevent the increases in serum creatinine and BUN associated with cisplatin-induced injury (130). Interestingly, this study also investigated the role of neutrophils in the context of enhanced injury. The authors had previously reported that depletion of CD11c+ cells worsens cisplatin-associated injury (114). Thus, they performed a double-depletion of CD11c+ cells and neutrophils to determine if neutrophils were responsible for the additional level of injury. Again, though, they saw that neutrophil depletion provided no benefit.

An additional study that may provide evidence against a role for neutrophils in ischemic injury investigated the role of the NLRP3 inflammasome. NLRP3 is a major player in organizing the inflammatory response in a variety of conditions and helps coordinate the processing and production of molecules like IL-1β and IL-18. NLRP3 also has a role in promoting pyroptosis (131). While NLRP3-deficient mice had no protection from cisplatin-induced AKI, they were protected to some degree from ischemia (132). This protection from IRI was observed despite no change in neutrophil recruitment to the kidney. This observation merits further investigation to explore nuances in AKI pathogenesis. If neutrophils are indeed involved in the development of AKI in certain settings, it is possible that NLRP3 does not impact neutrophil trafficking but plays a role in disease-promoting properties of neutrophils or directly protects kidney cells from death via inhibiting pyroptosis. In short, while there is evidence against the involvement of neutrophils in AKI, there is also an array of data that indicates they play a role in multiple settings. It is clear our knowledge in this arena is still incomplete and there is a need for further, careful investigation.

### F4/80+ Myeloid Cells: Macrophage, Monocyte, or Dendritic Cell?

The importance of infiltrating F4/80+ cells to AKI pathogenesis has proven a challenging element to resolve due to difficulties distinguishing between recruited monocytic cells and resident macrophage or DC populations. The distinction between macrophages and monocytes is often not clearly made and many studies conflate these populations. Further, there is frequent ambiguity about the delineation between DC, monocytes, and macrophages in the literature. This is a major source of confusion within the AKI field and has hindered our ability to draw precise conclusions.

Many methods for investigating the role of macrophages in disease also impact the monocytic or DC compartment in some fashion. As stated above, clodronate liposome, CD11b-DTR, and CD11c-DTR depletion models all have the capacity to deplete monocyte and DC populations, among others. Studies that manipulate trafficking signals such as integrins and selectins may also impair monocyte trafficking and other leukocytes in addition to the target cells of a given study.

Recent advances and development of new tools will hopefully allow clearer descriptions of distinct MPC populations' contributions during AKI. For example, it is now known that classical dendritic cells and their precursors express the ZBTB46 transcription factor while other myeloid lineages do not (94). Exploiting this discovery revealed that, indeed, only a small proportion of kidney-resident immune cells are DC (74, 94). This discovery has also led to the generation of ZBTB46-DTR mice for the specific depletion of DC (133), but this tool has yet to be used in models of AKI. The distinctions between monocytes and macrophages are still poorly defined since monocytes can transition into macrophages within tissue. Shared markers and closely related differentiation pathways continue to make it difficult to separate the biological contributions of macrophages and monocytes in living systems. Thus, limited means for specifically depleting or sequestering monocytes and macrophages has hindered investigations of these cells and their respective roles in AKI. Continued progress in this area will likely require the use of creative experimental systems, such as parabiotic models in which the skin of two mice is sutured together to allow for shared vasculature and circulating cells. Studies by Park et al. (79) and Lever et al. (71) have recently used this model and differential replacement kinetics following depletion to nicely investigate the role resident macrophages and monocyte/monocyte-derived macrophages in AKI. Their work showed that resident macrophages are minimally renewed by circulating cells, specifically express the V-domain Ig suppressor of T cell activation (VISTA) marker, and play an important role in recovery and repair following ischemic injury. These are powerful tools for distinguishing between resident and infiltrating cells in a given tissue. Use of additional markers to classify populations and subpopulations will also be beneficial for assessing myeloid cells present during kidney injury and some progress has been made in this regard.

An investigation of Tamm-Horsfall Protein's (THP) impact on macrophage regulation assessed myeloid subpopulations by sub-setting based on CD11b and MHC II (134). THP-deficient mice had lower proportions of CD11b^hi^ myeloid cells at baseline while a CD11b^mid^ MHC II^hi^ population was unchanged. The authors referred to the CD11b^hi^ cells as macrophages, but they more likely represent monocytes or monocyte-derived macrophages that have infiltrated the kidney (73, 74). Conversely, the CD11b^mid^ population most likely represents resident macrophages. Thus, there appeared to be a specific defect in monocytic cells in the absence of THP while resident cells remained constant.

IRI in the THP-deficient setting revealed that THP-deficient mice had greater increases in kidney neutrophils but reduced accumulation of the CD11b^hi^ Ly6G^lo^ cells (presumably monocytic lineage) cells. THP-deficient mice also showed greater neutrophil presence in steady-state kidneys prior to IRI. Interestingly, macrophages in THP-KO mice were less phagocytic, as demonstrated by impaired uptake of liposomes. Logically, it is possible that impaired macrophage phagocytic activity could result in impaired clearance of debris and damaged cells and lead to continuous, low-level inflammation and neutrophil recruitment, but this will require further investigation. It has been shown, though, that lack of the EPO receptor on macrophages leads to impaired clearance of apoptotic cells and age-dependent immune cell infiltration and kidney disease (135); an observation that could support this hypothesis. Another striking aspect of the THP-deficient mice was a notable lack of colony stimulation factor-1 (CSF-1, also referred to as M-CSF) after AKI. CSF-1 is an important growth factor for the differentiation of macrophages from precursors and monocytes. This may in part explain the preferential accumulation of neutrophils over monocyte-derived macrophages.

AKI was not extensively assessed in this model, but serum creatinine steadily increased in THP-deficient mice post-IRI through the 72-h analysis period. Reconstituting THP-deficient mice with a bolus of exogenous THP at 24 h post-IRI produced a transient reduction in serum creatinine which then began to increase again by 72 h, presumably as the effect of the bolus diminished. This indicates a role for THP in dampening injury by potentially supporting monocyte differentiation and macrophage phagocytic function. However, it is also interesting to note that diminished monocyte recruitment in the absence of THP did not prevent increases in creatinine. This raises questions about the role of F4/80+ cell accumulation in AKI pathogenesis.

Studies investigating the role of heme oxygenase-1 (HO-1) in AKI have also used additional markers to assess myeloid populations (136, 137). HO-1 is an enzyme that is induced in response to the accumulation of free heme. Heme is an iron containing compound that normally associates with multiple proteins to form hemoproteins that regulate a wide variety of biological processes. In contrast to its beneficial role in hemoproteins, free heme that is not bound to a functional protein can catalyze the formation of reactive oxygen species, a major source of cell stress and damage during IRI (138). HO-1 is the rate-limiting enzyme for the conversion of free heme into biliverdin, carbon monoxide, and free iron (Fe^2+^) and thus plays crucial cytoprotective roles in multiple tissue-injury settings (139, 140). Reperfusion following a period of ischemia can lead to an excess of free heme in the reperfused tissue and is a likely source of cellular toxicity during AKI.

A study performed with global HO-1-deficient mice employed the CD11b, MHC II, and F4/80 markers to assess myeloid subpopulations in greater detail (136). Mild ischemia (bilateral IRI, 10 min of ischemia) in global HO-1-deficient mice resulted in 60% mortality by day 2 post-IRI but was entirely sublethal in control mice, which highlights the important protective effects of HO-1. In the HO-1-deficient mice, kidney injury observed at day 1 post-ischemia was associated with a large increase in renal neutrophil representation (CD11b^hi^ MHC-II^lo^ Ly6G^hi^). The actual numbers of total monocytes/macrophages (CD11b+ MHC II+) were not closely examined and the overwhelming predominance of the neutrophil population made it difficult to evaluate potential increases or decreases in this population based on percentages alone. However, within the CD11b+ MHC II+ population, there were two readily distinguishable subpopulations: CD11b^hi^ F4/80^mid^ and CD11b^mid^ F4/80^hi^. The authors referred to the F4/80^hi^ population as DC, but the majority of this population has been shown to represent macrophages (74, 94). As referred to in Table 1, only a subset of DC expresses the F4/80 marker and this subset's expression level is lower than that of macrophages. The CD11b^hi^ cells are most likely monocyte-lineage cells that infiltrate and accumulate post-injury. Again, cell numbers of these 2 populations pre- and post-injury were not assessed, but HO-1-deficient mice exhibited an obvious skewing toward the CD11b^hi^ population when compared to WT mice. Thus, the absence of HO-1 leads to dramatic increases in neutrophilic inflammation and disruption of the macrophage compartment, even after mild ischemia.

The differences between global HO-1-deficient and WT mice and the importance of the observed disturbances in the myeloid compartments merit further investigation. HO-1 deficiency has dramatic impacts on tissue macrophage populations and the impacts of this during AKI are not yet understood. For example, macrophages in the spleen and liver are essentially depleted in HO-1-deficient mice and renal macrophages basally express high levels of the haptoglobin receptor CD163 (141). The skew toward CD11b^hi^ F4/80^mid^ cells could indicate basal fragility of resident macrophages and continual replacement by monocytes. It is still not fully known if adult monocyte-derived macrophages have phenotypic and functional alterations compared to true tissue-resident populations. Thus, if the majority of macrophages within the HO-1-deficient kidney are monocyte-derived, there is a possibility that their behavior during disease progression is altered. More detailed analysis of the resident cells in HO-1-deficient and WT mice will provide further insight into the phenotypic properties of tissue macrophages and their potential for promoting beneficial or detrimental processes.

Another study examined the role of HO-1 specifically in myeloid cells using a LysM-Cre conditional HO-1-knockout (137). These mice lack HO-1 expression specifically in LysM-expressing populations (predominantly macrophages, neutrophils, and monocytes) and do not suffer from the same degree of sensitivity to ischemia as global HO-1-deficiency. Thus, the mice were able to tolerate 26 min of IRI. While this ischemic time was not lethal, myeloid deletion of HO-1 was sufficient to cause increased levels of plasma creatinine and tubular injury in response to IRI when compared to littermate controls. Myeloid populations were broken into populations based on CD11b and F4/80 expression. Three populations were identified: CD11b^hi^ F4/80^lo^ (classified as neutrophils due to high Ly6G expression), CD11b^hi^ F4/80^mid^ (likely monocytes/monocyte-derived cells), and CD11b^mid^ F4/80^hi^ (mostly resident macrophages). Similar to global HO-1 deletion, IRI led to dramatic increases in neutrophils at 24 h post-injury with essentially no changes detected in the other populations. However, HO-1 expression was significantly upregulated in the non-neutrophil populations. Thus, HO-1 expression in macrophage/monocyte populations constitutes an important protective response during kidney injury.

The authors investigated this further using a hemin pretreatment protocol. The hemin molecule is nearly identical to heme, with the major difference being hemin contains ferric iron (Fe^3+^) rather than ferrous iron (Fe^2+^). Hemin is a potent inducer of HO-1 and the authors exploited this ability to increase HO-1 levels prior to inducing injury. Pretreatment with hemin prevented increases in plasma creatinine and specifically increased HO-1 expression in the CD11b^hi^ F4/80^mid^ population following IRI. Interestingly, accumulation of the CD11b^hi^ F4/80^mid^ population in kidneys 24 h after IRI was also enhanced by the hemin treatment. Therefore, HO-1 induction, specifically in monocytic populations, may be an important anti-inflammatory element of renal protection during injury. This is also interesting due to the fact that HO-1 is induced in myeloid cells by reno-protective cholinergic stimulation (142, 143).

Several questions regarding HO-1 and myeloid cells still remain. Since global HO-1-KO mice are extraordinarily susceptible to injury and death and have basally disrupted macrophage populations, this extreme model may not be ideal for investigating nuanced questions in the future. It will be interesting to know how heme release and HO-1 expression evolve during IRI in WT mice that progress to lethal disease. Are heme processing and scavenging systems overwhelmed in the setting of severe or sustained injury? In addition, the monocyte compartment contains multiple subpopulations. These studies did not investigate if HO-1 is equally expressed by these subsets or the cells they may differentiate into within tissue. Additionally, the ability of HO-1 to modulate iron species could imply a connection with ferroptosis. Perhaps HO-1 provides support to the anti-ferroptotic activity of GPX4 during injury and helps lessen disease severity. This is an interesting line of inquiry with potential to yield increased understanding of AKI pathogenesis and promising therapies.

Previous work showed that manipulating chemokine receptors known to be involved in monocyte trafficking (CCR2, CX3CR1, CXCR4) can limit the increase in renal F4/80+ cells and provide protection during AKI (21, 144, 145). One of the studies on CCR2 and CX3CR1 included a nice assessment of surface marker expression to distinguish between resident and monocyte-derived populations and revealed that the drop in F4/80+ cell accumulation was due to prevention of monocyte infiltration (21). This work showed that removing the ability of cells to respond to either CCR2 or CX3CR1 was sufficient to prevent IRI-associated increases in serum creatinine within 24 h, thus implicating monocyte-derived cells in AKI and supporting a previous observation that the lack of CCR2 signaling was able to reduce ischemic kidney injury (145). The work with CXCR4 is interesting since this cytokine receptor provides homing and retention signals. Signaling through CXCR4 retains mature cells and precursors in the bone marrow and prevents their release into circulation (146–149). This study used a CXCR4 antagonist compound to interfere with this signal and found that, although circulating leukocytes were increased, myeloid infiltration of the kidney was decreased and injury was ameliorated (144). Thus, while blocking CXCR4 signals reduces bone marrow retention, it also limits homing to and infiltration of the kidney by inflammatory cells.

However, the role of monocytic cells in AKI remains unclear. The THP and HO-1 studies above seem to indicate that injury can progress without substantial monocyte infiltration and that monocytic cells may even contribute to protection. The trafficking studies, on the other hand, suggest that the prevention of homing to the kidney is an effective means to reduce injury. The absence of a tool for specifically depleting monocytes makes sorting out the impact of these cells a difficult and complex task which will require creative solutions. It should be noted, however, that neutrophils can also express CCR2, CX3CR1, and CXCR4 so any benefit attributed to reduced monocyte trafficking in studies involving these molecules could be due to additional effects on neutrophils (144, 150–153).

Overall, development of kidney injury is a heterogeneous process with multiple routes to disease. Different immune populations may play larger or smaller roles depending on the AKI-initiating event and this may be a source of confusion within the field. The involvement of neutrophils vs. monocytes requires further careful investigation to refine our knowledge base. The role of macrophages in recruiting these cells is still unsettled due to the inability of many models to specifically manipulate macrophages over other related cell types. There is likely a link between cell death and activation of resident populations, but there is much work to be done to prove this connection and define the role of additional myeloid populations that may be recruited via macrophage activation.

## Regulation Through CSF1R During AKI

The CSF-1 receptor (also known as Csf1R, CD115, and c-Fms) is critical for macrophage development. Disruption of the *Csf1r* gene results in a near complete lack of F4/80+ cells in adult tissues (154). These mice are also osteopetrotic due to the lack of oscteoclasts, which creates complications for assessing the impact of CSF1R on bone marrow-derived monocytes. Egress of these cells into circulation is diminished in *Csf1r*-deficient mice. However, blocking CSF1R later in life with antibody treatment allows analysis of this receptor's impact without interfering with development. This approach has revealed that CSF1R activity is not required for the production of monocytes but does regulate their subsequent differentiation (155, 156). As monocytes mature, they transition from a Ly6C^hi^ CX3CR1^lo^ phenotype to a Ly6C^lo^ CX3CR1+ phenotype (157). Blocking CSF1R leads to severe reductions in the Ly6C^lo^ subset in circulation and tissue. Blockade also prevents the reconstitution of tissue macrophages by peripheral monocytes. Thus, the CSF1R appears to govern the differentiation pathways of Ly6C^hi^ monocytes and the ability of monocytes to transition into macrophages.

The CSF1R has two distinct ligands that may differentially impact outcome during kidney injury. Knockout of one of these ligands, IL-34, resulted in lesser F4/80+ cells in the kidneys following IRI (158). In IL-34-sufficient mice, F4/80+ cells were slightly increased by day 1 post-IRI and further accumulated at days 3 and 5. IL-34-deficient mice showed a similar pattern of accumulation but to a lesser overall degree at all time points. Reduced infiltration of F4/80+ cells was accompanied by lower levels of kidney injury molecule-1 (KIM-1), serum neutrophil gelatinase-associated lipocalin (NGAL), and urine albumin during early injury. At 20 days post-IRI and later, IL-34-deficient mice exhibited less severe fibrosis and better preservation of kidney architecture. Thus, IL-34 signaling appears to promote an inflammatory environment that favors worse injury, potentially through its action on macrophages and monocytes. Although absence of IL-34 does not completely prevent the accumulation of F4/80+ cells, lack of this cytokine was still sufficient to improve outcome. This supports the concept that while increases in F4/80+ cells is an indicator of disease, the molecules and signals the cells experience in the kidney are also important for determining their contribution.

A role for colony stimulating factor-1 (CSF-1), the second CSF1R ligand, was investigated in IRI and DT models of kidney injury (111). As referred to above, the DT model of disease relies on expression of the human DTR under control of a proximal tubule specific promoter (*Ggt1* promoter in this case). Since murine cells are insensitive to DT, injection of DT results in toxicity specifically to tubule cells which results in injury and decreased kidney function.

CSF-1 knockout also limited the accumulation of F4/80+ cells in the kidney following injury, reminiscent of the IL-34 deficient mice. Although the IL-34- and CSF-1-deficient models cannot be directly compared here, the degree of residual F4/80+ accumulation appeared greater in IL-34-deficient mice. Despite the potentially more profound lack to F4/80+ accumulation, the absence of CSF-1 signaling worsened early injury and delayed recovery. This directly contrasts with the protection observed with IL-34-deficiency. This study also probed the involvement of macrophage/dendritic cell (DC) populations during injury by performing depletions with clodronate and CD11c-DTR models. In both depletion strategies, the lack of macrophage/DC prevented overall F4/80+ cell accumulation in the kidney, but also worsened injury. Altogether, the data from these studies point to complex and opposing functions of monocyte/macrophage populations during kidney injury. The phagocytic and debris clearing properties of macrophages may be important for ameliorating the extent of initial injury. Macrophage-mediated inflammation in response to death of tissue cells, on the other hand, appears to drive the accumulation of additional F4/80+ cells (and likely neutrophils). The recruited myeloid populations may then contribute to either injury or recovery, depending on the signals they receive. According to the data currently available, IL-34 promotes proinflammatory activity and delayed recovery while CSF-1 skews cells toward tissue support and injury resolution phenotypes. Interestingly, tubules are a major source of CSF-1 in the kidney (112). This begs the question of whether tubule injury and death reduces the overall concentrations of CSF-1 by removing a cellular source of the cytokine and skews the environment toward IL-34 signaling—thereby limiting the beneficial functions of macrophages and promoting inflammation. These interesting findings merit additional, detailed studies to directly compare the impact of these molecules on monocyte/macrophage dynamics, phenotypic profile, and function in various models of kidney injury.

## Discussion

Clearly, the involvement of cell death, macrophage activation, inflammation, and immune cell infiltration in AKI disease is complex. Our understanding is steadily improving, but there are still key unknowns that need to be addressed. There is strong evidence for the importance of necrotic death pathways in promoting injury and disease, but different modes of death may have greater or lesser roles in specific disease models and may even work in concert during the development of injury. The impact of these death pathways on resident macrophages is an important knowledge gap that must be resolved, but anecdotal evidence suggests a connection between inflammatory death, immune recruitment, and disease severity which could center around macrophage activation.

However, depletion models also indicate macrophages may be involved to varying degrees depending on the injury model used. This divergence could also relate to the death pathways at play and we should work toward a unified understanding of injury models, death pathways, and macrophage/myeloid involvement. This information would be invaluable for properly assessing AKI patients and developing adaptive/adaptable treatment strategies. Given the diverse data that has been published in this field, it is important to remember that AKI represents a syndrome that can manifest from distinct insults that may produce variations on a theme of defining characteristics. It is likely that different manifestations will require more tailored therapies to address root causes and key differences.

**Figure 3:**
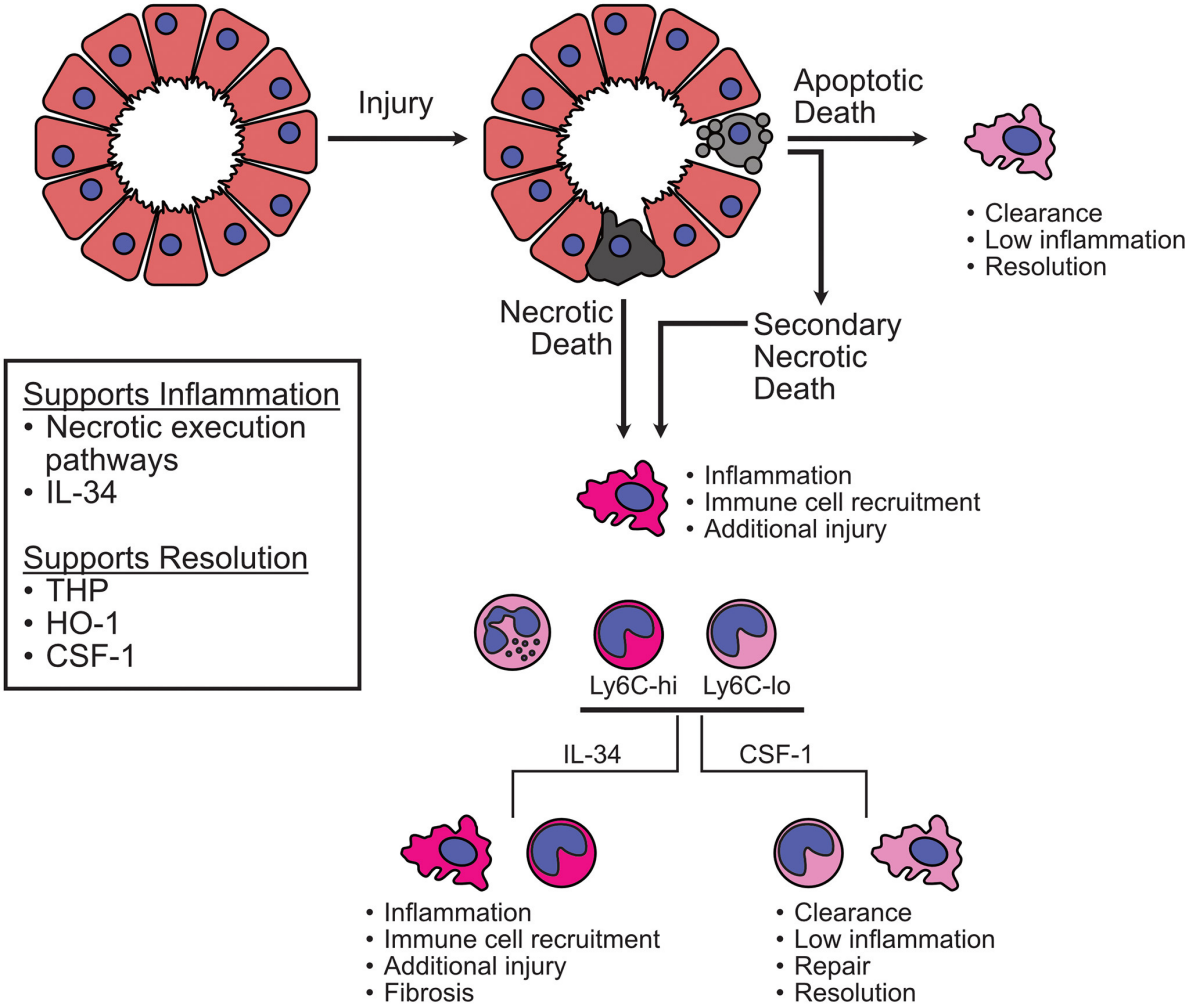
Potential macrophage/monocyte responses to initial injury and regulation via CSF1R during AKI. After initial injury, cell death can occur via multiple pathways. Apoptotic cells can be cleared by efferocytosis and results in relatively low inflammation. If clearance is insufficient to resolve the injury, more inflammatory forms of cell death may pre-dominate, such as necroptosis or necrosis. Based on the evidence discussed in this review, inflammatory cell death can activate resident macrophages to produce proinflammatory molecules and recruit additional cells. This leads to an influx of neutrophils and monocytic cells. Monocytes can transition into additional macrophage populations whose activity is determined by signals from the environment. According to the studies discussed here, IL-34 supports more proinflammatory activity while CSF-1 supports more reparative activity. Interfering with the necroptotic pathway of cell death lessens injury-induced inflammation and promotes recovery. THP appears capable of enhancing the phagocytic function of macrophages, which supports clearance and resolution. HO-1 allows for the breakdown of toxic free heme to lessen injury and inflammation. Overall, a balance must be struck between interrupting progressive inflammation and injury while supporting clearance of damaged material and repair.

Further investigation of myeloid populations is also warranted. The relevance of neutrophils to disease is still not clear despite many studies investigating their involvement. Again, injury model and cell death contexts should be carefully considered when evaluating the impact of neutrophils. Data shows that macrophages/monocytes are relevant to processes that contribute to both tissue damage and support. The dynamic nature of these cells and their ability to integrate environmental stimuli does not preclude contradictory roles, but defining the critical elements that dictate their impact during AKI will be a delicate endeavor requiring sophisticated experimental design. As the data stands now, beneficial processes appear to revolve around proper debris/dead cell clearance and prevention of excessive cell death. For example, the ability of THP to enhance macrophage phagocytic activity, HO-1 to limit heme-mediated ROS production and iron availability, and RIPK deficiency/blockade to reduce inflammatory cell death (Figure 3). Detrimental elements appear to stem from unrestrained inflammation in the context of sterile cell death. This is illustrated by the continuous accumulation of myeloid cells from early injury through later stages of disease. From this point of view, it is possible that IL-34 activity is designed to induce anti-pathogen activity in monocytes/macrophages and induction of this cytokine in response to danger signals results in the accumulation of an inappropriate cell type that causes additional damage in the absence of a pathogen to attack (Figure 3). Overall, additional studies employing sophisticated means of delineating the specific impacts of macrophages, monocytes, and DC are needed. The rise of high-dimensional single cell analysis techniques is making headway toward this goal and should yield interesting, informative data in the near future (70, 159, 160). We have entered a new era with exciting tools emerging that will help address these issues and provide much needed answers. More detailed analysis of important death pathways and myeloid populations in the context of AKI will enable pursuit of innovative and targeted treatment options for AKI patients. Ultimately, we must continue to ensure that our strategies for research analysis and therapy development are sufficiently advanced to address the complexity inherent to the AKI syndrome. This is chess, not checkers.

## Author Contributions

WN wrote and prepared the manuscript, table, and figures with input and revision from MO.

## Conflict of Interest

The authors declare that the research was conducted in the absence of any commercial or financial relationships that could be construed as a potential conflict of interest.
